# Longitudinal Neuropsychological Profile in a Patient with Triple A Syndrome

**DOI:** 10.1155/2013/604921

**Published:** 2013-04-09

**Authors:** Luigi Mazzone, Valentina Postorino, Lavinia De Peppo, Lia Vassena, Laura Fatta, Marco Armando, Giuseppe Scirè, Marco Cappa, Stefano Vicari

**Affiliations:** ^1^Child Neuropsychiatry Unit, Department of Neuroscience, I.R.C.C.S. Children's Hospital Bambino Gesù, Piazza S. Onofrio 4, 00165 Rome, Italy; ^2^Endocrinology Unit, I.R.C.C.S. Children's Hospital Bambino Gesù, Piazza S. Onofrio 4, 00165 Rome, Italy

## Abstract

Triple A syndrome is an autosomal recessive disorder characterized by the triad of adrenocorticotropic hormone resistant adrenal insufficiency, achalasia, and alacrima. Our aim was to describe the neuropsychological characteristics and the cooccurring psychopathological and neurological disorders in an Italian male child suffering from Triple A syndrome at the time of admission (T0) and after one year of follow-up (T1). Many difficulties were observed in the motor domain, as well as in manual dexterity and static/dynamic balance domains of the motor task over time. In sharp contrast with previous literature reports on frequent mild cognitive dysfunction in patients with Triple A syndrome, our child did not show any mental retardation. By contrast, he showed an average IQ at T0 with a slight improvement at T1. To our knowledge, this report is the first describing neuropsychological profile and co-occurring psychopathological problems in a child with Triple A syndrome. Considering that the Triple A syndrome is a progressive disorder which can take years to develop the full-blown clinical picture, these patients require periodical medical controls. Moreover, assessment of neuropsychological and psychopathological features should be performed in patients with this disease, in order to underline the variability of this syndrome.

## 1. Introduction 

Triple A syndrome is as an autosomal recessive disorder characterized by the triad of adrenocorticotropic hormone (ACTH) resistant adrenal insufficiency, achalasia, and alacrima [1–3]. The first manifestation is usually alacrima [4–11]. Achalasia appears with advancing age in 75% of patients [3]. Adrenal insufficiency normally arises later in life developing gradually over the first decade [12], but in some cases hypoglycemia and seizures may also occur as presenting symptoms contributing to the diagnosis of the disease. In addition to these three main features, many patients also show additional clinical manifestations including neurological abnormalities such as progressive peripheral polyneuropathy, hyperreflexia, nasal speech, developmental delays, and dysautonomia, often associated with mild mental retardation [13].

Genetic linkage analysis revealed a locus on chromosome 12q13 that is commonly mutated in individuals with Allgrove syndrome [14] and, subsequently, the gene called AAAS encoding for a protein of 547 amino acids, called ALADIN (alacrima/achalasia/adrenal insufficiency/neurologic disorder), was identified as the site of mutations in Allgrove syndrome [15, 16].

Although disabling neurologic manifestations often lead to neuropsychological impairments, to our knowledge, no previous reports have described the neuropsychological profiles in these patients. The aim of our paper is to describe the longitudinal neuropsychological characteristics and the co-occurring psychopathological disorders of an Italian boy with a clinical diagnosis of Triple A syndrome.

## 2. Case Presentation

### 2.1. Medical History

A male patient (child; aged 5, 6 years) was admitted to the Child Neuropsychiatry Unit of the Children's Hospital Bambino Gesù of Rome (Italy) for neurological and cognitive assessment. The patient was the second child of nonconsanguineous parents, born at term with a natural childbirth after a pregnancy with miscarriages during the third month. Birth weight was normal (3,280 grams). The neonatal period was regular, with maternal nursing. Autonomous walking occurred at 15-16 months of age. First words were spoken at 12-13 months. Parents referred a regression in motor and relational skills at the age of 14 months, since when the patient showed repeated episodes of dyspnea, vomiting, and abdominal pains with swallowing difficulties. At the age of two years old, the child was diagnosed with achalasia. Alacrima was present in the child since birth, and at the age of four years old the Schirmer's test was performed showing a positive result with a reduced tear production bilaterally in the eyes. Moreover, ACTH stimulation test and cortisol levels showed adrenocortical insufficiency with high level of plasma ACTH (>1250 pg/mL) and low level of serum basal cortisol (1.64 ng/mL), and substitution therapy was started. In light of these results, he was diagnosed with Triple A syndrome (achalasia, alacrimia, and addison). Soon thereafter he started to show epileptic seizures with loss of consciousness and tonic/clonic contractions lasting a few minutes, even though EEG, a brain MRI, and brain CT returned normal results. He also experienced hypoglycemic episodes and in October 2010 he was subjected to an esophageal myotomy. 

A complete neurological, neuropsychological, and psychopathological assessment was performed at the time of the admission (age 5, 6 years) (T0), and after one year (T1), over a period of three days every time. Between T0 and T1 the patient practiced psychomotor and speech therapy four times a week. 

### 2.2. Genetic Examinations

Genetic examination revealed a compound heterozygous AAAS mutation consisting of two mutations. One allele carried a heterozygous frameshift mutation: a T deletion at position 429 (c.429delT) causing a frameshift after phenylalanine 143 resulting in a nonsense sequence of 28 amino acids followed by a premature stop codon (p.Phe143fs or F143fs). The other allele carried a heterozygous missense mutation in exon 12: a T > G transversion at position 1142 (c.1142T > G) resulting in a substitution leucine → arginine at position 381 (p.Leu381Arg or L381R).

### 2.3. Longitudinal Neurological Assessment

At the time of admission (T0), the child showed a mild muscle weakness with a reduction of tendon reflexes and a mild impairment in coordination with an intrarotation of the feet during the cerebellar tests. Indeed, he was unable to stand on toes. Neurophysiologic examinations showed a predominant axonal motor and sensory neuropathy with a delayed central motor function. The same neurological symptomatology was observed after one year (T1).

### 2.4. Intelligence Evaluation

Cognitive evaluation was performed at the time of admission (T0), and after one year (T1) using Wechsler preschool and primary scale of intelligence—third edition (WPPSI-III) [17]. At T0 the patient showed an average cognitive profile (Total Intelligence Quotient (TIQ) = 88) with a higher score in the verbal area (Verbal Intelligence Quotient (VIQ) = 98) and a lower score in the nonverbal one (Performance Intelligence Quotient (PIQ) = 85). At T1 the same performance was observed in cognitive evaluation, but with a slight improvement: the patient showed an average cognitive profile (TIQ = 91) with a higher score in the verbal area (VIQ = 96) and a lower score in the nonverbal one (PIQ = 91) (Table 1).

### 2.5. Neuropsychological Assessment

At T0 to evaluate the adaptive level of the patient the semistructured Vineland Adaptive Behavior Scales (VABS) [18] interview was completed by parents with an experienced and trained clinician. The child scored below average on all four adaptive areas (communication, daily living skills, socialization, and motor skills) indicating poor personal skills. He also showed problems in visual tests, performing poorly in sustained visual attention on the Bells Test [19]. Moreover, he reported a great difficulty in motor tests, such as in the overall Developmental Test of Visual-Motor Integration (DVMI) [20], with a lower score in Motor Coordination subtest. In line with this, the child also had many difficulties in performing selected motor items, including “stand on one foot” and “toe walking,” resulting in manual dexterity, ball skills, and static/dynamic balance below average of the Movement Assessment Battery for Children (MABC) [21]. On the evaluation of the verbal short-term memory the child revealed an average performance for his age in the direct test of “Battery for the assessment of neuropsychology for developmental age” [22] and in the “Memory and learning tasks for developmental age” Nonword Repetition task [23], whereas in the reverse test of BVN [22] the child's performance has not been possible to evaluate, apparently due to difficulties in understanding the task itself. Similarly, at T0 the child's planning skills have not been evaluated because he was not able to perform the corresponding test “Tower of London” (TOL) [24].

At T1, although the parents reported a better adaptive level in all areas of VABS [18], the patient continued to obtain lower scores than expectations for chronological age. He also continued to show great difficulty in motor tests (i.e., DVMI and MABC) [20, 21] and in verbal short-term memory reverse task (i.e., BVN) [22] whereas an average performance in verbal short-term memory direct tasks (i.e., BVN and Nonword Repetition) [22, 23].

Contrarily, the child showed an improvement in the performance on the Bells Test [19] in both selective and sustained visual attention and in the planning skills being able to complete the TOL task [24], although with a performance below average.

Neuropsychological assessment is shown in Table 1.

### 2.6. Psychopathology Evaluation

At T0 the psychopathology evaluation, using Child Behavior Checklist (CBCL 1,5–5) [25], revealed clinical scores in internalizing problems, anxious problems, and pervasive developmental problems, as well as somatic problems and attention problems. These results were confirmed by clinical scores in the following items of Conners' Parent Rating Scale-Revised (CPRS-R) [26]: cognitive problems/inattention, hyperactivity, ADHD index, and inattention. By contrast, according to Swanson Rating Scale-IV (SNAP-IV) [27] and Scale for the Individuation of Attention Deficit and Hyperactivity Disorders (SDAG) [28], the child's performance was on average.

After one year (T1) the child showed a significant improvement in all the CBCL and CPRS-R areas that at the time of admission resulted in the clinical range, although the CBCL internalizing and somatic problems continue to reveal borderline sores. These results were confirmed by the average scores of the SNAP-IV and SDAG.

Psychopathology evaluation is shown in Table 2.

## 3. Discussion

The present study describes an Italian boy diagnosed with Triple A syndrome. We focused our assessment on his neuropsychological characteristics and co-occurring psychopathological and neurological disorders during one year. Although patients with Triple A syndrome have highly heterogeneous clinical features [29], it is possible to identify a similar pattern and progression [30], with symptoms and manifestations that usually appear in a timeline highly similar to that observed in this patient. 

The child did not show any mental retardation even after one year of follow-up and this finding is in sharp contrast with previous literature reports on frequent mild cognitive dysfunction in patients with Triple A syndrome. For instance, Vallet et al. [31] documented intellectual disability in five out of six patients, ranging from slight (three patients) to moderate (one patient) mental retardation and in other reports the IQ was shown to gradually decrease over time [29]. In line with this last observation, one possible explanation for the inconsistency documented in this case report could be related to the very young age of the patient at the time of admission. However, after one year of follow-up we did not find a decrease in IQ, by contrast a slight improvement. It was noticed that in the paper by Grant et al. [32] two brothers with Allgrove syndrome have been followed and thoroughly reevaluated (case 1 from age 3 to 15 and case 2 mainly at the age of 14 years) in both cases the cognitive profiles and other clinical features were identical over time. 

Conversely, the child showed an adaptive level lower than his chronological age (both T0 and T1), characterized by difficulties in daily functioning and everyday living skills, including walking, talking, getting dressed, going to school, and preparing a meal. Many difficulties were also observed in the motor domain, as assessed by DVMI, as well as in two out of the three domains (manual dexterity and static/dynamic balance) of the motor task, and were stable over one year. The child showed many difficulties in tasks as “stand on one feet” and “toe walking”, that have been consistently described as first signs of motor difficulties in Triple A syndrome and have been reported for other cases in the literature [33]. A significant deficit in planning competence was also found at the time of admission: during the evaluation the experimenter verified that the child was actually capable of understanding the instructions of the test, which included a practice phase, but he was not able to practice the test. However, at the follow-up the child was able to complete the task although his performance remained below average for chronological age, once again highlighting his motor difficulties (mainly in fine motor skills).

Concerning attention abilities, at T0 the results presented herein documented a borderline performance on the visual selective attention domain and a score significantly pathologic in the visual sustained attention domain. During the T0 assessment the child was inattentive and hyperactive, not being able to complete independently a task, and he was easily distractible, despite being willing to cooperate, in line with the psychopathology evaluation that revealed clinical scores in domains such as attention problems or hyperactivity. By contrast, after one year, during the follow-up, the child has not shown these attention and hyperactive deficits during the assessment. This is confirmed by his average performance on the visual selective and sustained attention and by the average scores found in the psychopathology evaluation.

Finally, adrenal insufficiency is one of the three broad symptoms of Triple A syndrome. According to literature data, patients affected by adrenal insufficiency (Addison's disease) show increased levels of anxiety and a higher risk for affective and mood disorders [34–36]. Confirming these data, at the time of admission the psychopathology assessment of our patient revealed CBCL clinical scores in internalizing problems, anxious problems, pervasive developmental problems, and somatic problems. These problems continued to be reported at the follow-up with the exception of anxious problems. In conclusion, to our knowledge, this report is the first describing a neuropsychological profile and cooccurring psychopathology problems in a child with Triple A syndrome. Considering that the Triple A syndrome is a progressive disorder [35, 36] which can take years to develop the full-blown clinical picture, these patients require periodical medical controls. As mental retardation and other multisystem neurological symptoms can arise over time [30, 31], assessment of neuropsychological and psychopathological features should be performed in patients with this disease, in order to underline the variability of this syndrome and the need for a multidisciplinary approach.

## Figures and Tables

**Table 1:** Longitudinal cognitive and neuropsychological assessment.

Examined skill	T0	T1
	At admission	After one year
Cognitive level		
WPPSI-III—Wechsler preschool and primary scale of intelligence	TIQ^a^: 88; VIQ^b^: 98; PIQ^c^: 85	TIQ: 91; VIQ: 96; PIQ: 91
Adaptive level		
VABS—Vineland Adaptive Behavior Scales:		
Communication	a.e.^d^ 3, 7 years	a.e. 5, 4 years
Daily living skills	a.e. 2, 11 years	a.e. 4, 1 years
Socialization	a.e. 3, 1 years	a.e. 4, 5 years
Motor skills	a.e. 3, 1 years	a.e. 4, 4 years
Visual-Motor Integration		
DVMI—Developmental Test of Visual-Motor Integration:		
Visual-Motor Integration	Rs^e^: 6; Ss^f^: 67; a.e. 3, 6 years; *P* ^g^: 1°	Rs: 6; Ss: 62; a.e. 3, 6 years; *P*: 1°
Visual-Motor Integration Visual Test	Rs: 13; Ss: 95; a.e. 5, 1 years; *P*: 37°	Rs: 13; Ss: 88; a.e. 5, 1 years; *P*: 21°
Visual-Motor Integration Motor Test	Rs: 8; Ss: 73; a.e. 3, 5 years; *P*: 4°	Rs: 10; Ss: 78; a.e. 4, 1 years; *P*: 7°
Attention		
Bells Test:		
Selective visual attention	<1,9 SD^h^	<0,9 SD
Sustained visual attention	<2,3 SD	<0,9 SD
Memory		
BVN—Battery for the assessment of neuropsychology for developmental age		
Verbal short-term memory:		
Direct	Span: 3; <0,5 SD	Span: 3; <1,3 SD
Reverse	Not evaluable	Not evaluable
PROMEA—Memory and learning tasks for developmental age		
Nonword Repetition	Rs: 32; *P*: 75°	Rs: 31; *P*: 50°
Planning		
Tower of London	Not evaluable	Rs: 14; *P*: <5°
Motor skills		
MABC—Movement Assessment Battery for Children:		
Manual dexterity	Rs: 9,5; *P*: 5°	Rs: 11; *P*: <5°
Ball skills	Rs: 0; *P*: >15°	Rs: 6; *P*: <5°
Static and dynamic balance	Rs: 12,5; *P*: <5°	Rs: 13,5; *P*: <5°

^a^Total Intelligence Quotient.

^b^Verbal Intelligence Quotient.

^c^Performance Intelligence Quotient.

^d^Age-equivalent score.

^e^Raw score.

^f^Standard score.

^g^Percentile.

^h^Standard deviation.

**Table 2:** Longitudinal psychopathology evaluation.

Psychopathology	T0 At admission	T1 After one year
CBCL—Child Behavior Checklist 1.5–5 and 6–18		
Competence scales:		
Activities	—	Ss^a^: 22 C^c^
Total competence	—	Ss: 22 C
Syndrome scale:		
Emotionally reactive	Ss: 69 B^b^	—
Anxious/depression	Ss: 66 B	Ss: 53
Somatic complaints	Ss: 72 C	Ss: 68 B
Attention problems	Ss: 70 C	Ss: 59
Internalizing, externalizing, total problems, and other problems:		
Externalizing problems		
Internalizing problems	Ss: 69 C	Ss: 61 B
Total problems	Ss: 67 C	Ss: 56
DSM-oriented scales:		
Affective problems		Ss: 65 B
Anxiety problems	Ss: 73 C	Ss: 60
Pervasive developmental problems	Ss: 70 C	—
Attention deficit/hyperactivity problems	Ss: 67 B	Ss: 53
CPRS-R—Conners' Parent Rating Scale Revised		
Cognitive problems/inattention	Ss: 73 C	Ss: 47
Hyperactivity	Ss: 70 C	Ss: 48
ADHD index	Ss: 80 C	Ss: 50
Restlessness/impulsivity	Ss: 65 B	Ss: 51
Clinical global index total	Ss: 62 B	Ss: 51
Inattention (DSM IV)	Ss: 70 C	Ss: 48
DSM IV total	Ss: 63 B	Ss: 45
SNAP-IV—Swanson Rating Scale-IV		
Inattention	Rs^d^: 10; Ss: 1,1	Rs: 10; Ss: 1,1
Hyperactivity/impulsivity	Rs: 12; Ss: 1,3	Rs: 4; Ss: 0,4
Combined ADHD	Rs: 22; Ss: 1,2	Rs: 14; Ss: 0,7
Oppositional-defiant disorder	Rs: 10; Ss: 1,2	Rs: 6; Ss: 0,75
SDAG—Scale for the Individuation of ADHD^e^		
Inattention	Rs: 10; Ss: 1,1	Rs: 9; Ss: 1
Hyperactivity	Rs: 9; Ss: 1	Rs: 3; Ss: 0,3
Combined inattention/hyperactivity	Rs: 19; Ss: 1,05	Rs: 12; Ss: 0,6

^a^Standard score.

^b^Borderline score.

^c^Clinical score.

^d^Raw score.

^e^Attention Deficit and Hyperactivity Disorder.
